# Supplementary material for: Impaired Myofibroblast Proliferation is a Central Feature of Pathologic Post-Natal Alveolar Simplification
Source: bioRxiv. 2024 Sep 16:2023.12.21.572766. Originally published 2023 Dec 23. Preprint. [Version 2] doi: 10.1101/2023.12.21.572766 (PMC10769348; doi:10.1101/2023.12.21.572766)

bioRxiv preprint doi: https://doi.org/10.1101/2023.12.21.572766; this version posted September 16, 2024. The copyright holder for this preprint (which was not certified by peer review) is the author/funder, who has granted bioRxiv a license to display the preprint in perpetuity. It is made available under aCC-BY 4.0 International license.

Supplementary Figure Legend

Supplementary Figure 1. scRNA-seq of Murine Lungs With Neonatal Hyperoxia Treatment or Loss of Epithelial TGFβ Signaling. (**A**) UMAP projection of all scRNA-seq data. Outlined in red are the mesenchymal cell populations. (**B**) UMAP plots showing expression levels of canonical markers for epithelial, endothelial, hematopoietic, mesenchymal, and mesothelial populations. (**C**) Total cell number within each of the indicated major lung populations. (**D**) Differentially expressed genes in myofibroblasts were identified by comparing either CTRL RA vs O2 cells or RA CTRL vs cKO cells. These lists were subsequently analyzed by Qiagen IPA to identify predicted upstream regulators for each comparison. The Venn-diagram on left depicts the number of overlapping predicted upstream regulators with z-score <-1.5 (upregulated), while the table on right lists these 40 shared upstream regulators.

Supplementary Figure 2. Characterization of Mesenchymal Cell Clusters by scRNA-seq. (**A**) UMAP projection of mesenchymal cells from scRNA-seq data as outlined in Figure S1. (**B**) Heatmap of the top ten most differentially expressed genes across mesenchymal clusters. The intensity of expression is indicated as specified by the color legend. (**C**) UMAP plots showing expression levels of select canonical markers for alveolar myofibroblast, ductal myofibroblast, Col13a1 fibroblast, and Col14a1 fibroblast populations as depicted by recent work by Hurskainen *et al*. and Narvaez Del Pilar *et al*. (**D**) Dot plot showing selected markers for each cluster within the mesenchyme.

Supplementary Figure 3. Re-analysis of Published Data Confirms Loss of Myofibroblasts With Neonatal Hyperoxia Treatment. (A) Hurskainen *et al.* treated C57BL/6 wildtype mice with 85% hyperoxia versus normoxia from P0-P14 and analyzed the lungs by scRNA-seq at P3, P7, and P14 [47]. The Seurat object used for publication was provided by the authors. UMAP projection shows the mesenchymal populations as defined by Hurskainen *et al.* (**B**) By using metadata within the Seurat object, we graphed the frequency of each mesenchymal population by treatment condition and time

bioRxiv preprint doi: https://doi.org/10.1101/2023.12.21.572766; this version posted September 16, 2024. The copyright holder for this preprint (which was not certified by peer review) is the author/funder, who has granted bioRxiv a license to display the preprint in perpetuity. It is made available under aCC-BY 4.0 International license.

point. (**C**) The frequency of myofibroblasts within the mesenchyme as depicted in (B). **(D)** Xia *et al.* treated C57BL/6 wildtype mice with 85% hyperoxia versus normoxia from P0-P14 and analyzed the lungs by scRNA-seq at P14 [50]. The Seurat object used for publication was provided by the authors. UMAP projection shows the mesenchymal populations as defined by Xia *et al.* (**E**) By using metadata within the Seurat object, we graphed the frequency of each mesenchymal population by treatment condition and time point. (**F**) The frequency of alveolar and ductal myofibroblasts within the mesenchyme as depicted in (E). Graphs in (C) and (F) depict one value for each condition because we were unable to extract replicate values from the data provided.

Supplementary Figure 4. Gli1-CreERT2 **Allele Does Not Disrupt Alveolar Development, But Pdgfra**-CreERT2 **Allele Worsens Hyperoxia-induced Injury.** (**A**) Either Gli1-CreERT2 or Pdgfra-CreERT2 mice and their cre-negative littermates were injected with tamoxifen on P2 and P4, treated in 75% hyperoxia versus normoxia from P0-P10, and recovered in room air until harvest at P40 for analysis by histology. (**B**) Mean linear intercepts of Gli1$^{CreER/+}$ and Gli1$^{+/+}$ mice treated as outlined in (A) and harvested at P40. (**C**) Mean linear intercepts of Pdgfra$^{CreER/+}$ and Pdgfra$^{+/+}$ mice treated as outlined in (A) and harvested at P40. Data compared by ANOVA with Fisher's post hoc test. Error bars depict mean ± SEM. ***p<0.001, ****p<0.0001.

Supplementary Figure 5. Loss of TGFβ Signaling to Lung Mesenchyme Causes Worse Disease in Hyperoxia While Itgb6 Plays No Role in Alveolar Development. (**A**) Tgfbr2$^{F/F}$ and Tgfbr2$^{F/F}$;Gli1-CreERT2 littermates were injected with tamoxifen on P2 and P4, treated in 75% hyperoxia versus normoxia from P0-P10, and recovered in room air until harvest at P40 for analysis by histology. (**B**) H&E sections of representative lungs from (A) harvested at P40 (left). Mean linear intercepts calculated for all treatment groups (right). (**C**) Itgb6$^{F/F}$ and Itgb6$^{F/F}$;Nkx2.1-cre littermates were treated in 75% hyperoxia versus normoxia from P0-P10, and recovered in room air until harvest at P40 for analysis by histology (**D**) H&E sections of representative lungs from (C) harvested at P40 (left). Mean linear

bioRxiv preprint doi: https://doi.org/10.1101/2023.12.21.572766; this version posted September 16, 2024. The copyright holder for this preprint (which was not certified by peer review) is the author/funder, who has granted bioRxiv a license to display the preprint in perpetuity. It is made available under aCC-BY 4.0 International license.

intercepts calculated for all treatment groups (right). Data compared by ANOVA with Fisher's post hoc test. Error bars depict mean ± SEM. *p<0.05, ***p<0.001, ****p<0.0001. Scale bars = 100 μm.

Supplementary Figure 6. Decreased PDGFRα Mean Fluorescence Intensity Across Multiple Models of Alveolar Simplification. (**A**) Mean fluorescence intensity (geometric mean, MFI) of PDGFRα antibody staining on MCAM-negative mesenchymal cells (CD45-, CD31-, Epcam-) and PDGFRa+ cells (CD45-, CD31-, Epcam-, MCAM-, PDGFRα+) as quantified by flow cytometry. Each column represents an experiment shown earlier in this study: normoxia vs hyperoxia at P10 (Figure 2), Tgfbr2$^{F/F}$ vs Tgfbr2$^{F/F}$;Nkx2.1-cre at P10 (Figure 2), PBS vs 1D11 at P10 (Figure 8), Ect2$^{F/F}$ vs Ect2$^{F/F}$;Pdgfra-CreERT2 at P14 (Figure 9). To compare values across multiple experiments, MFI's were normalized to the control condition within each experiment. Data compared by ANOVA with Fisher's post hoc test. Error bars depict mean ± SEM. ***p<0.001, ****p<0.0001.

bioRxiv preprint doi: https://doi.org/10.1101/2023.12.21.572766; this version posted September 16, 2024. The copyright holder for this preprint (which was not certified by peer review) is the author/funder, who has granted bioRxiv a license to display the preprint in perpetuity. It is made available under aCC-BY 4.0 International license.

Supplementary Figure 1

A

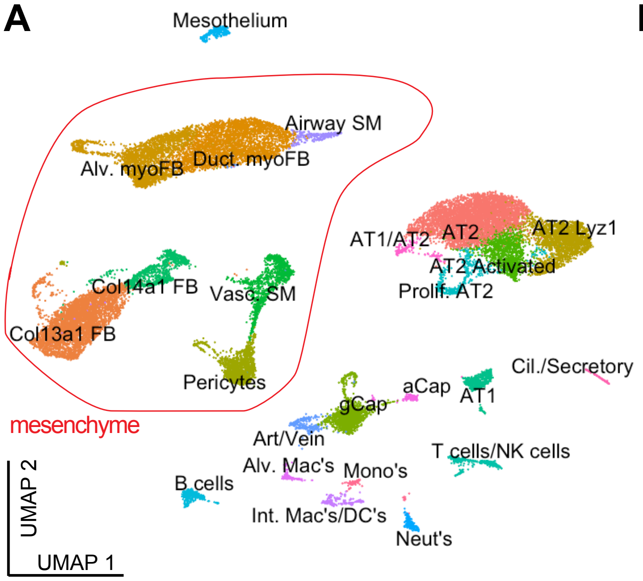

B

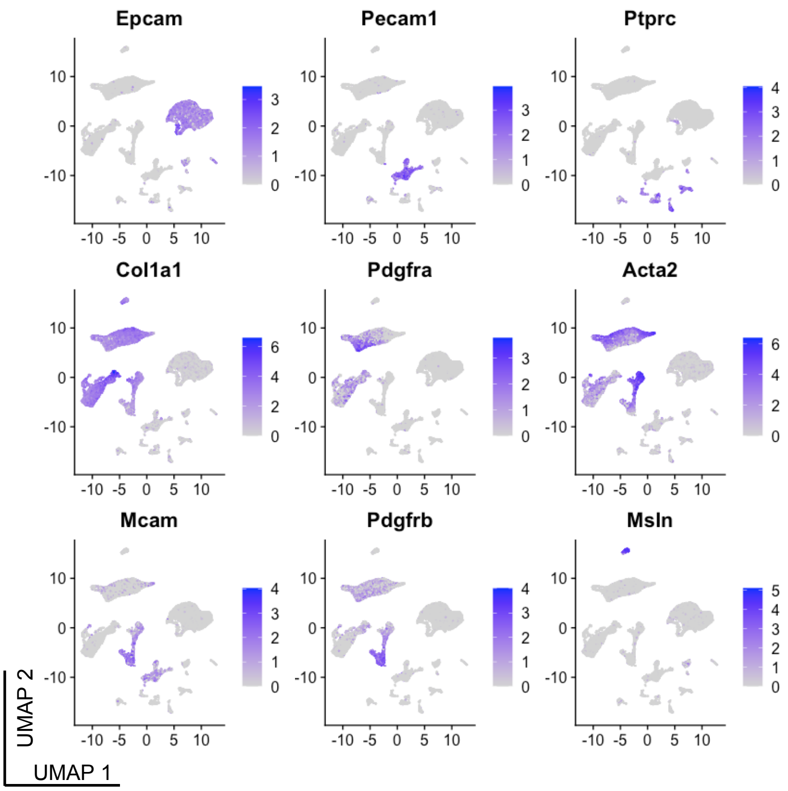

C

Subset	Cells
Mesenchyme	13,331
Epithelium	9,262
Endothelium	1,856
Hematopoietic	1,798
Mesothelium	363
**Total**	**26,610**

D

Myofibroblast IPA:
z-score <-1.5 (Downregulated)

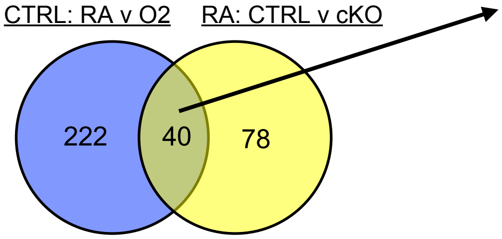

Shared Predicted Upstream Regulators (Downregulated)

ADAM12	GNAS	MEF2C	PPARG
ALDH1A2	GPER1	miR-30c-5p	RELA
APEX1	HDAC2	NANOG	SMAD7
BDNF	HGF	NCOA2	TLR3
COL5A1	IFNG	NFkB (complex)	TNF
CREB1	IL1B	NGF	TSC2
CTNNB1	LEP	NOD2	Vegf
ELK1	LIF	NRAS	
EPHA2	MAP2K5	PDGF BB	
FSH	MAP3K8	PDGFB	
GLI3	MAPK1	POMC	

bioRxiv preprint doi: https://doi.org/10.1101/2023.12.21.572766; this version posted September 16, 2024. The copyright holder for this preprint (which was not certified by peer review) is the author/funder, who has granted bioRxiv a license to display the preprint in perpetuity. It is made available under aCC-BY 4.0 International license.

Supplementary Figure 2

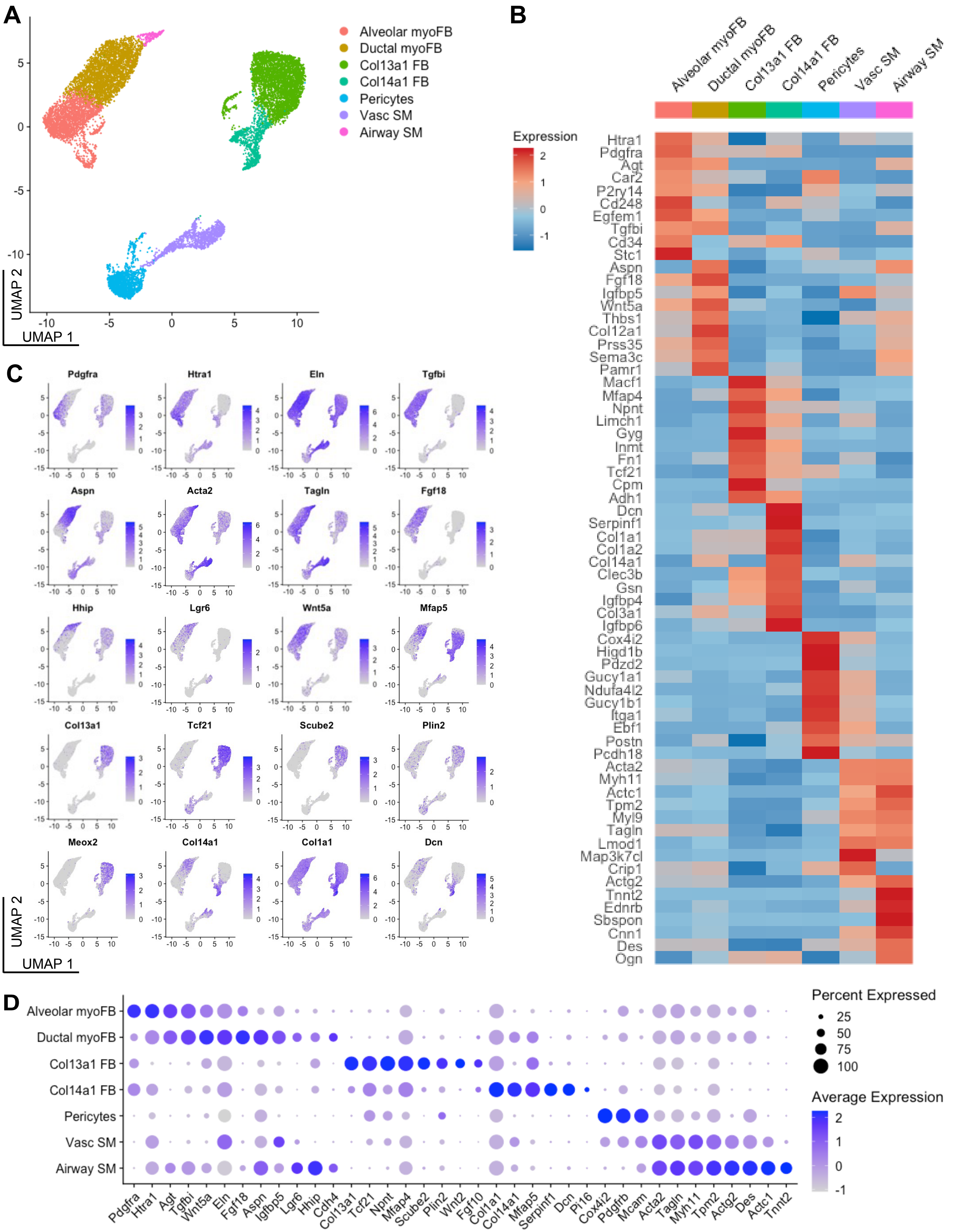

bioRxiv preprint doi: https://doi.org/10.1101/2023.12.21.572766; this version posted September 16, 2024. The copyright holder for this preprint (which was not certified by peer review) is the author/funder, who has granted bioRxiv a license to display the preprint in perpetuity. It is made available under aCC-BY 4.0 International license.

Supplementary Figure 3

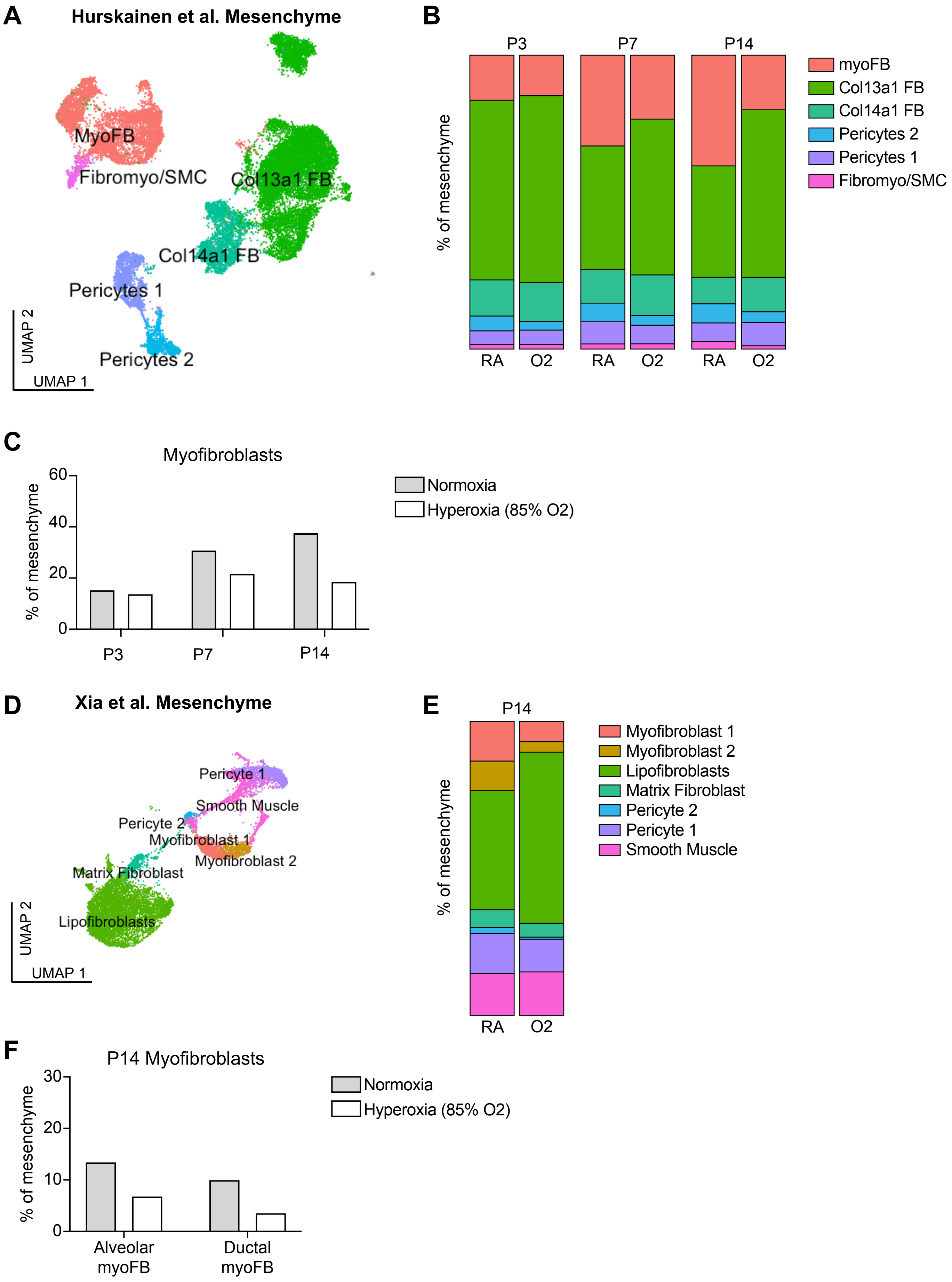

bioRxiv preprint doi: https://doi.org/10.1101/2023.12.21.572766; this version posted September 16, 2024. The copyright holder for this preprint (which was not certified by peer review) is the author/funder, who has granted bioRxiv a license to display the preprint in perpetuity. It is made available under aCC-BY 4.0 International license.

Supplementary Figure 4

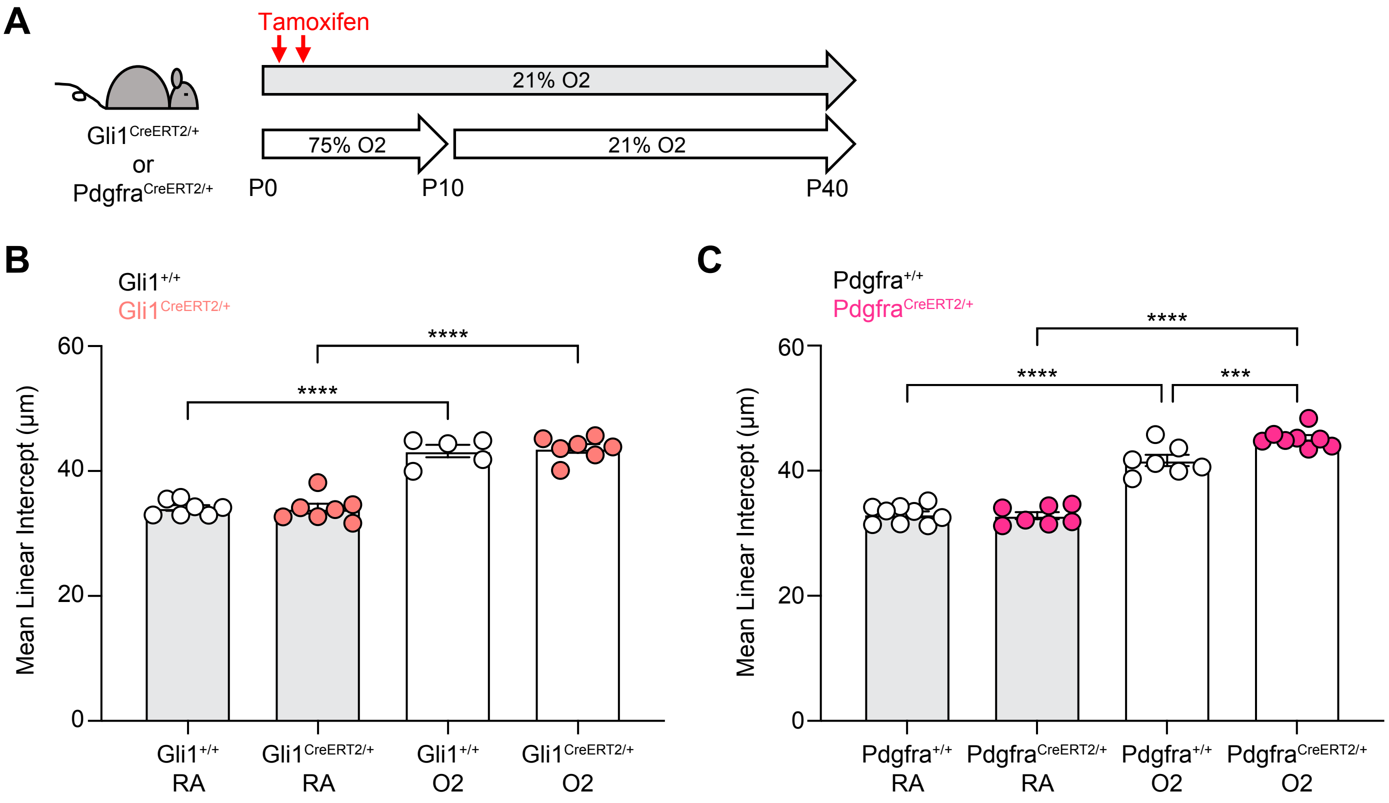

bioRxiv preprint doi: https://doi.org/10.1101/2023.12.21.572766; this version posted September 16, 2024. The copyright holder for this preprint (which was not certified by peer review) is the author/funder, who has granted bioRxiv a license to display the preprint in perpetuity. It is made available under aCC-BY 4.0 International license.

Supplementary Figure 5

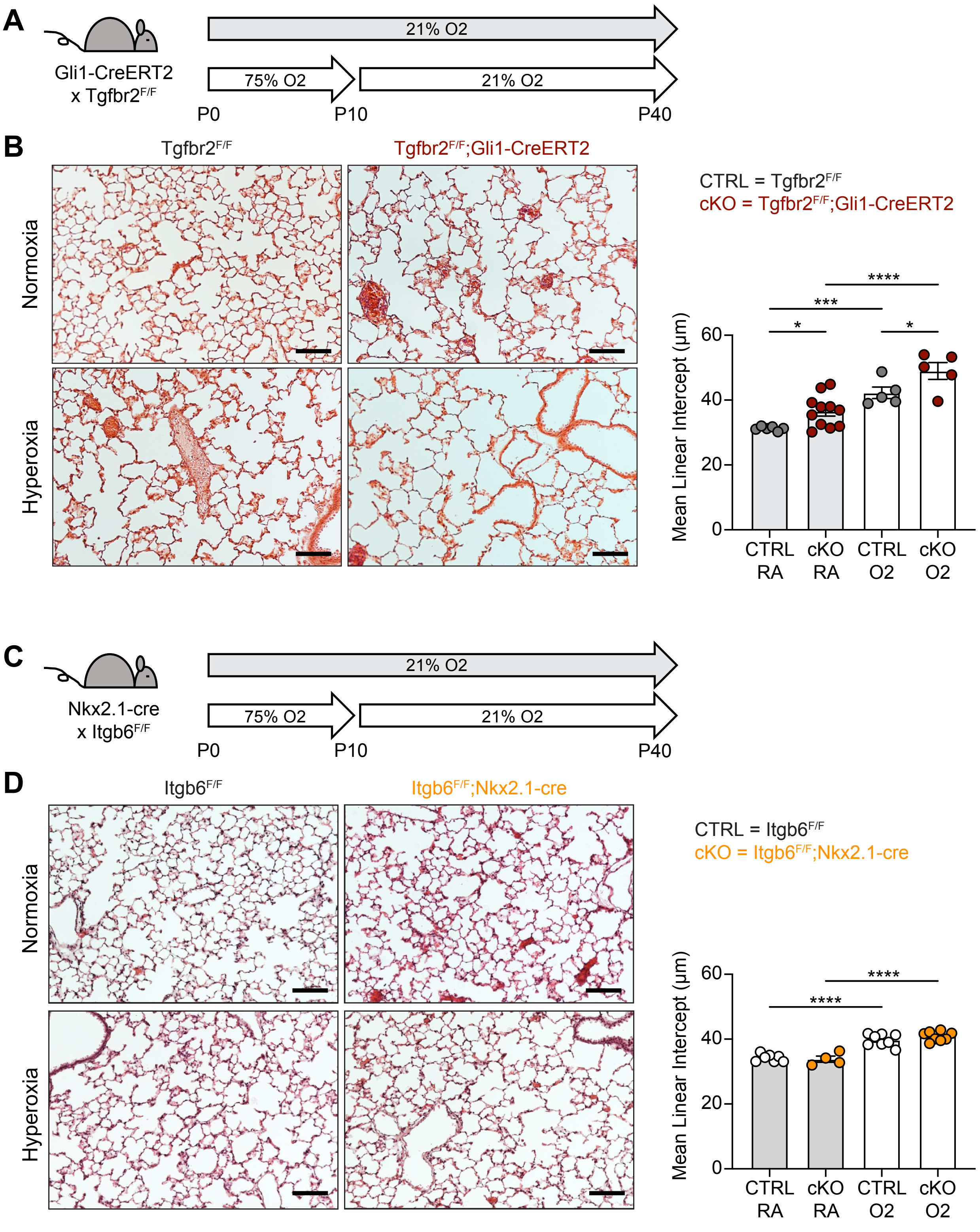

bioRxiv preprint doi: https://doi.org/10.1101/2023.12.21.572766; this version posted September 16, 2024. The copyright holder for this preprint (which was not certified by peer review) is the author/funder, who has granted bioRxiv a license to display the preprint in perpetuity. It is made available under aCC-BY 4.0 International license.

Supplementary Figure 6

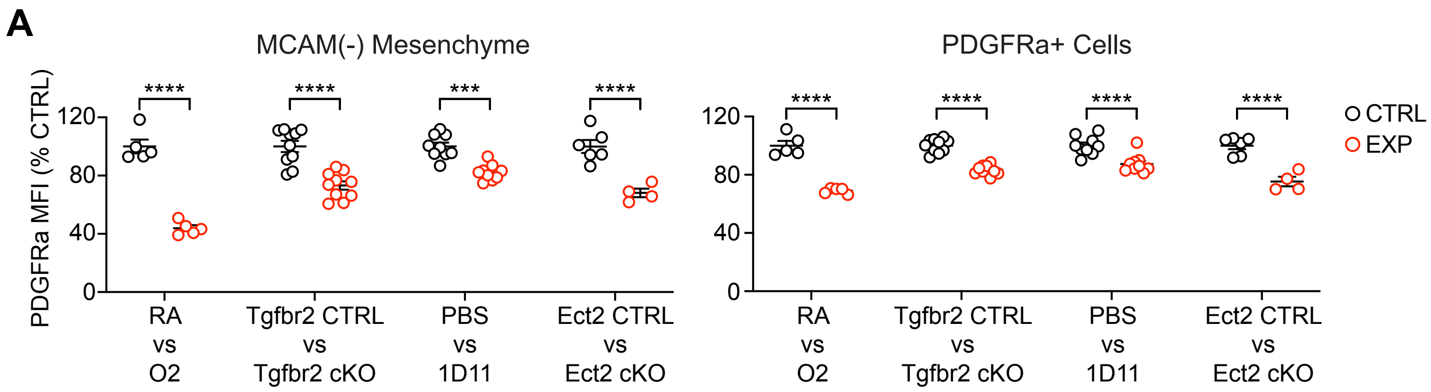

Supplement: 1 [file NIHPP2023.12.21.572766V2-supplement-1.pdf]
